# Qualitative and biochemical characteristics of pomegranate fruit grown using reclaimed water and low input fertigation treatments at harvest and during storage

**DOI:** 10.1016/j.heliyon.2024.e34430

**Published:** 2024-07-14

**Authors:** Michela Palumbo, Virginia Carbone, Ilde Ricci, Bernardo Pace, Maria Cefola, Paola Minasi, Simone Pietro Garofalo, Salvatore Camposeo, Anas Tallou, Gaetano Alessandro Vivaldi

**Affiliations:** a Institute of Sciences of Food Production, National Research Council of Italy (CNR), c/o CS-DAT, Via Michele Protano, 71121 Foggia, Italy; b Institute of Food Sciences, National Research Council of Italy (CNR), Via Roma, 64 83100 Avellino, Italy; c Department of Agricultural and Environmental Sciences, University of Bari Aldo Moro, Via Amendola 165/a, 70126 Bari, Italy

**Keywords:** *Punica granatum* L., Water reuse, Respiration rate, Bioactive compounds, Polyphenolic compounds

## Abstract

In recent years, severe climate change leading to by water scarcity reduced water quality has increased the need for effective irrigation strategies for agricultural production. Among these, the reuse of reclaimed water represents a non-expensive and reliable solution. The effect of conventional or reclaimed water, applying convention or smart fertigation system, were investigated during two irrigation seasons on yield, qualitative and biochemical traits of pomegranates fruit (cv Wonderful One) at harvest, and after storage at 7 °C. The results of this study showed that using reclaimed waters with different fertigation systems did not affect the pH values, total soluble solids, and titratable acidity on pomegranates fruit showing slight decrease changes only during postharvest storage. On the other hand, the respiration rate was not affected by water quality. Furthermore, the antioxidant activity was also preserved during storage in pomegranates fruit from plants irrigated with reclaimed water by applying conventional or smart fertigation. The analysis also identified 52 compounds by UHPLC-MS^n^ and HPLC-UV-Vis analyses. A slight decrease (about 17 %) at harvest and during storage in polyphenols content was shown in fruit grown using reclaimed water. The study demonstrates that using reclaimed water is a sustainable and effective way to limit the use of conventional water for irrigating pomegranate crops without significant reduction in yield, or in qualitative and nutritional values of the fruit at harvest and during storage.

## Highlights

• Reclaimed water resulted in higher quality than conventional one.
• Reclaimed water is suitable for pomegranate irrigation.
• Pomegranate quality and physiological injuries are not affected by RW during storage.
• Total polyphenols content was significantly affected by treatment and storage time.

## 1 Introduction

Nowadays, climate change represents one of the most challenging problems at the global level that must be faced and solved, as previously reported during the World Economic Forum in 2018 [1]. An increase of 20 % in the number of areas and people affected by droughts has been recorded among the years 1976–2006 in the European Union as a direct consequence of climate change [2]. In addition, climate change, unpredictable weather patterns and droughts contribute significantly to the strain on freshwater availability, arising from urban development and agriculture [3]. To support the lack of water in terms of quality and quantity for agricultural productions, the EU issued a regulation on minimum requirements for water reuse in irrigation, which entered into force in June 2020 and came into effect in June 2023 [3].

The Florida Department of Environmental Protection defines reclaimed water (RW) as - water that has received at least secondary treatment and basic disinfection and is reused after flowing out of a domestic wastewater treatment facility [4]. Furthermore, using RW could potentially reduce the need for supplemental applications of mineral fertilizer and it is considered non-expensive and reliable, particularly for irrigation in agriculture [5]. So, the use of RW appears particularly interesting in all geographical contexts characterized by scarcity of irrigation water and no optimal rainfall with irrigation lands, as in many regions in Southern Mediterranean countries.

In Europe, water reuse constitutes a priority to mitigate the impacts of climate change, especially water scarcity. Approximately 1 Mm^3^ of RW is annually repurposed, representing around 2.4 % of the total treated urban wastewater effluents, yet constituting less than 0.5 % of the annual freshwater withdrawals in the EU. Projections suggest that by 2025, the volume of RW will reach 3222 Mm^3^/year in Europe [6]. Spain is considered the first country in terms of RW reuse, with an annual volume of 347 hm^3^/year, mainly used in agricultural practices, mirroring global trends. Specifically, 71 % of the RW volume is allocated to crop irrigation, 17 % to environmental purposes, 7 % to recreational activities, and 4 % to urban applications, while merely 0.3 % is appropriated for industrial usage [7]. Nevertheless, the adoption of this strategy is still in progress due to the absence of environmental and health safety regulations, fluctuation in RW quality and the presence of different hazardous compounds. The potential presence of microorganisms, potentially toxic elements, compounds of emerging concern, and microplastics in RW can present health risks and environmental threats and further evaluative researchers are crucial [8].

Among these, the Apulia region in the South of Italy is characterized by hot and dry summers, which require a high volume of irrigation water for permanent crops and different species of vegetables that cover over 80 % of irrigation lands [9]. Different studies were conducted on fruit tree crops using RW for irrigation in this region. For example [10], studied the impact of irrigating almonds with saline RW and desalinated water under full irrigation and regulated deficit irrigation strategies. They highlighted that a combination of RW and regulated deficit irrigation was a fruitful practice for almond trees. In another experiment, the same Authors, also evaluated the physiological responses of almond trees under the same conditions using remote sensing technology [11], confirming the positive impact of RW as a strategy for sustainable agriculture and water management. Moreover [[12], [13], [14]], applied RW on different crops (i.e. tomato, grapefruit and olive trees) in an arid region (Murcia, Spain) and the results in terms of yield, growth, and fruit quality were higher than the crops irrigated with freshwater.

In the last five years, pomegranate (*Punica granatum* L.) has one of the most representative permanent crops, which increased the production surface by over 100 %, [15]. The leading cultivar worldwide is represented by the Wonderful, discovered in Florida and brought to California 100 years ago characterized by large-sized sweet-and-sour fruit containing red arils and ripening at the beginning of October [16,17]. Although pomegranate trees are considered drought-tolerant and their cultivation is suitable for semi-arid regions, this crop requires irrigation during dry summer to obtain optimal yield and fruit and juice quality parameters [18]. The pomegranate is considered a non-climacteric fruit for its physiologic characteristics, low levels of respiration rate and ethylene production [19]. Harvest time is commonly assessed based on skin color and acidity but the acceptance by the consumer is correlated with a combination of several quality attributes that are related to the physicochemical and mechanical properties including skin color, absence of physical defects, sugar content, acidity, and flavor [20]. Moreover, for the fresh market, the large size of the pomegranate fruit is an important aspect that is particularly appreciated by consumers as well as the geometric characteristics in terms of width and length [21,22].

Pomegranate fruit are indicated as a superfood, since are rich in bioactive compounds such as phenolic acids, flavonoids, vitamins, organic, sugars, antioxidants, and mineral elements [23,24]. The richness of these compounds is affected by cultivar, climatic conditions, ripening stage, growing and storage conditions [[25], [26], [27], [28]]. In pomegranate, over 90 % of the antioxidant activity of the whole fruit is covered by two types of polyphenolic compounds: anthocyanins (such as delphinidin, cyanidin, and pelargonidin) which give the red color to skin, juice, and hydrolysable tannins (such as punicalin, pedunculagin, punicalagin, gallagic and ellagic acid esters of glucose) [29,30]. It has been highlighted that punicalagin is the principal substance responsible for the total antioxidant capacity of juice, while anthocyanins play only a secondary role [31,32]. Fruit quality is preserved by applying specific postharvest handling conditions and practices such as storage temperature, relative humidity and suitable packaging [33], specifically for the pomegranate fruit cv Wonderful, the minimum safe storage temperature recommended, until 2 months is 7 °C, with high relative humidity (90–95 %) is to reduce weight loss is suggested [34].

To our knowledge, however, nothing has been published about the irrigation effects on pomegranate tree cv Wonderful One with RW. Therefore, this work intends to assess the effects of the use of RW on (i) pomegranate fruit yield and (ii) pomegranate fruit quality at harvest and after storage at 7 °C until 45 d, in two consecutively crop cycles. This work comes as a response to the European Green Deal strategy to reach a sustainable economy and environment by turning different challenges into new opportunities.

## 2 Materials and methods

### 2.1 Plant material

The trial was performed during two irrigation seasons (2020 and 2021) in an experimental orchard located out-doors in a plot of the University of Bari “Aldo Moro” located in the southeast of Italy (Bari, Apulia Region) (41°06′41″, 16°52′57″ E, 5 m a.s.l). Three years old pomegranate trees (*Punica granatum* L. cv Wonderful One) were cultivated in 350 L polyethylene pots (Height 70 cm, upper diameter 85 cm, lower diameter 70 cm, and a working volume of 250 L) filled with approximately 400 kg of soil with a texture classified as loam (44.78 % sand, 12.32 % clay, and 42.90 % silt). The pots planting system was 2 m between trees in a row and 3 m between rows oriented from N-NE to S-SW. Pomegranates, grown using different irrigation water sources, conventional water (CW) and RW, were provided for two consecutive years. RW was obtained using an on-field pilot plant, located in the Campus of the University of Bari, developed to treat municipal wastewater. The plant consisted of 2 storage tanks of 10 m^3^/each, a centrifugal pump that sent, at a constant flow rate, the municipal wastewater to a batch reactor with a volume of 2.5 m^3^, in which 4 UV lamps were installed and three chemicals were dosed: oxidant, sulfuric acid, and antiscalant. Therefore, the treatments were performed simultaneously with the help of a slow agitator to improve the diffusion of the chemicals. A battery of 4 UV lamps of 80 W each was installed inside the reactor to make oxidation effective. UV acted as a catalyst for the oxidizing power of the chemical substance used. Upon exiting the reactor, a centrifugal pump extracted a constant flow rate and sent it to a 10 m^3^ storage tank in which sulfuric acid connected to a pH-meter was dosed (since an increase in the pH in the reactor was expected). The pomegranate trees were subjected to three different treatments using CW or RW and applying conventional or smart fertigation as following reported: conventional water + conventional fertigation (CW-CF), reclaimed water + conventional fertigation (RW-CF), and reclaimed water + smart fertigation (RW-SF). For each treatment, there were available 12 pomegranate plants in randomized blocks design, whereas both years, the source, and the quantity of fertilizers (N, P, and K) are shown below in Table 1. When considering the total nutrients input (fertilizers + irrigation water), RW-CF consistently had the highest total nutrient. Pest control practices and pruning were commonly used by growers in the area, and no weeds were allowed to develop in the pots.

**Table 1:** The fertigation plan for 2020 and 2021 applied for the pomegranate trees.

Year	2020				2021		
Source of nutrients (kg ha^−1^)		Fertilizers	Irrigation water	Total	Fertilizers	Irrigation water	Total
N	CW-CF	57	1	57	82	1	83
	RW-SF	34	22	56	39	42	81
	RW-CF	57	22	79	82	42	124
P	CW-CF	53	0	53	69	0	69
	RW-SF	45	4	49	59	4	63
	RW-CF	53	4	53	69	4	73
K	CW-CF	50	2	52	90	9	99
	RW-SF	30	21	51	70	22	94
	RW-CF	50	21	71	90	22	112

Plants were irrigated according to the crop coefficient method's daily crop evapotranspiration (ETc). ET_c_ was calculated using the following equation recommended by FAO:

(1) ET_c_ = Kr * Kc * ET_0_

Where K_r_ is the reduction coefficient (K_r_ = 0.75), K_c_ is the crop coefficient as reported by Ref. [35]. ET_0_ is the reference of evapotranspiration, and it was calculated by the Penman-Monteith equation using climate data provided by the climate station located 100 m from the experimental field. The water was supplied by drip irrigation with four pressure-compensated drippers per tree, each with a flow rate of 2 L h^−1^.

#### 2.1.1 Water quality assessment

The inorganic solute content, pH, and electrical conductivity (EC) of the two irrigation water sources (CW and RW) were assessed during the irrigation season from May to September. The samples were collected in glass bottles, transported in an ice chest to the laboratory, and stored at 5 ^°^C before being processed for chemical and physical analyses. The concentrations of Na^+^, K^+^, Ca^2+^, B^3+^, NH_4_^+^, and Mg^2+^ were determined by an inductively coupled plasma optical emission spectrometer (ICP-ICAP 6500 DUO Thermo, England). Anions (Cl^−^, F^−^, NO_3_^−^, PO_4_^3−^, SO_4_^2−^, Br^−^) and total phosphorus were analyzed by ion chromatography with a liquid chromatograph (Metrohm, Switzerland). EC was determined using a PC-2700 m (Eutech Instruments, Singapore), and pH was measured with a pH-meter Crison-507 (Crison Instruments S.A., Barcelona, Spain) [36,37].

#### 2.1.2 Dimensions and weight of pomegranate fruit

The equatorial and polar dimensions, respectively the width and length, were collected for all fruit of each tree and treatment, starting from the 22 August (day of the year, DOY = 235) and 2 September (DOY = 245) for the first and the second year respectively. Fruit measurements were conducted with a manual caliper, every 10 days until harvest time, and used for detecting the shape of pomegranate expressed as aspect ratio using the following formula [22]:

(2) $$\text{Aspect}\;\text{ratio}:\frac{a\;\left(length\;of\;fruit\;in\;cm\right)}{b\;\left(width\;of\;fruit\;in\;cm\right)}$$

The weight of fruit was measured at harvest using a digital balance KERN 440-35 N (Balingen, Germany) and each one was assigned to one of the three following weight classes: 200–300 g; 300–400 g; 400–500 g [38].

### 2.2 Postharvest experiment setup

After each year's harvest, the pomegranate fruits were transported to the postharvest laboratory of the Institute of Sciences of Food Production - CNR and stored at 7 °C for 45 d in polypropylene bags to limit dehydration. Just after harvest and at the end of the storage, samples of each treatment (about 10 fruit) were analyzed in triplicate for the following quality parameters: skin and juice color, total soluble solids, titratable acidity, maturity index, pH, respiration rate, and antioxidant activity as below reported.

Moreover, the fruits coming from the second year were subjected to a biochemical characterization in the laboratory of the Institute of Food Science (CNR), as detailed in the next paragraphs.

#### 2.2.1 Analysis of pomegranate quality parameters

##### 2.2.1.1 Color analysis

The color of pomegranates was acquired using a colorimeter (CR-400-Konica Minolta, Osaka, Japan) equipped with a D65 illuminant in the reflectance mode and the CIE L* a* b* color scale. The skin and juice color of the fruit were analyzed in triplicate (n = 3) for each treatment, at harvest (0 d), and at the end of the storage (45 d). The skin color was measured on 6 random points on the fruit surface, whereas the color of the juice, was measured in a dark glass for a total of three times for replicate.

The a* and b* color parameters were used for the calculation of the hue angle (h°) using the following formula:

(3) h° = arctg *b∗ a∗*^−1^

##### 2.2.1.2 Juice pH, titratable acidity, total soluble solids, maturity index, respiration rate, and total antioxidant activity

Pomegranate juice was extracted for each replicate and treatment using a squeezer (Juicer SZP 25 B2 - Silver Crest, Hamburg, Deutschland). Juice pH was measured using a pH meter (PH-Burette 24 -Crison Instrument, Barcelona, Spain). The same instrument was used to measure titratable acidity (TA) determined by acid-base potentiometry (0.1 mol L^−1^ NaOH up to pH 8.1). TA was expressed as a percentage (%) considering citric acid as a major one. The total soluble solid (TSS) content, was recorded using a digital refractometer (DBR35-XS Instruments, Carpi, Italy) at room temperature and results were expressed as °Brix. The maturity index (MI) was calculated as the ratio of TSS/TA.

The respiration rate of pomegranates was measured at 7 °C using a closed system, according to the method reported by Ref. [34]. Pomegranate fruit, (about 1 kg) for each irrigation treatment and replicate was put into a 3.6 L hermetic plastic jar (one jar per replicate, n = 3), where CO_2_ was allowed to accumulate up to 0.1 % of the standard concentration of the CO_2_.

The measure of CO_2_ concentration was conducted by injecting 1 mL of gas sample from the headspace of the plastic jars through and injecting it into a gas chromatograph (p200 micro-GC - Agilent, Santa Clara, CA, USA) equipped with a dual column system and a thermal conductivity detector. The respiration rate was reported as mL CO_2_/kg*h.

Total antioxidant activity was determined by following the 1-ldiphenyl-2-picrylhydrazyl (DPPH) method. For each replicate, 5 g of pomegranate juice was homogenized in 20 mL methanol/water solution (80:20 v/v) for 2 min, using a homogenizer (T-25 digital ULTRA-TURRAX® - IKA, Staufen, Germany). After that, the mixture was centrifuged (Prism C2500-R, Labnet, Edison, NJ, USA) at 6440 g for 5 min at 4 °C. The absorbance was measured at 515 nm after 40 min in the dark condition, using a spectrophotometer (UV-1800, Shimadzu, Kyoto, Japan).

The DPPH radical scavenging activity was determined by the below-given formula:

(4) ((A blank - A sample)/A blank) *100

Whereas A blank is the absorbance value of the control reaction containing all reagents except the sample. The free radical scavenging activity, determined by DPPH, was expressed as EC_50_ value. It is defined as the volume (μL) required to decrease the initial DPPH radical activity by 50 % [39].

#### 2.2.2 Biochemical analysis

##### 2.2.2.1 Chemicals

High-Performance Liquid Chromatography (HPLC) grade acetonitrile, formic acid, and acetone were obtained from Merck (Darmstadt, Germany). Standard phenolic compounds were purchased from Merck and Extrasynthese (Genay, France). All other chemicals and solvents used were of the research highest purity grade.

##### 2.2.2.2 Extraction of phenolic compounds

Samples of 5 g of intact arils, manually obtained from fruit, were treated with 7 mL of acetone, and crushed. Extraction was performed for 10 min on a horizontal shaker in the dark. After centrifugation at 4000 rpm for 10 min, the supernatant was removed, the pellet was suspended in 5 mL of 2 % formic acid in acetone and the extraction was carried out as described. After centrifugation at 4000 rpm for 10 min, the supernatant was removed, the pellet was suspended in 3 mL of acetone/water 70/30 and the extraction was carried out as described. This extraction was repeated twice. Finally, the supernatants were pooled and dried in a rotary evaporator (LaboRota 4000/HB Efficient, Heidolph, Schwabach, Germany) and stored at −20 °C until use.

##### 2.2.2.3 Analysis of total phenolic content

The total phenolic content in all pomegranate extracts was determined according to the Folin-Ciocalteau's protocol [40] using gallic acid as a reference standard. 62.5 μL of Folin-Ciocalteu's reagent and 250 μL of distilled water were added to 62.5 μL of suitable aqueous dilution of dry extracts. The reaction mixture was mixed and allowed to stand for 6 min. Finally, 625 μl of sodium carbonate and 500 μL of distilled water were added, and the solution was incubated in the dark for 90 min. Then, the samples' absorbance was measured at 760 nm. The results were expressed as mg gallic acid equivalents (GAE) per gram of fresh weight (fw) (mg GAE g^−1^ fw). All measurements were carried out in triplicates.

##### 2.2.2.4 UHPLC-MS^n^ analyses

Extracts of pomegranate samples were reconstituted in water and analyzed by ultra-high performance liquid chromatography (UHPLC)-linear ion trap mass spectrometry on an LTQ XL ion-trap mass spectrometer coupled with a UHPLC Ultimate 3000 RS chromatographic system equipped with a Diode Array Detector and Xcalibur® system manager data acquisition software (Thermo Fisher Scientific, Waltham, MA, USA). Individual compounds were separated on an XBridge BEH C18 column (130 Å, 5 mm, 2.1 mm × 150 mm, Waters Inc., Milford, CT, USA) at a flow rate of 200 μL min^−1^; solvent A was 0.1 % formic acid in acetonitrile and water (4:96, v/v) and solvent B was 0.1 % formic acid in acetonitrile and water (54:46, v/v). After a 2 min hold at 0 % solvent B, elution was performed by a linear gradient from 0 to 20 % solvent B in 26 min, from 20 to 40 % solvent B in 15 min, from 40 to 60 % solvent B in 5 min and from 60 to 95 % solvent B in 5 min, followed by 10 min of maintenance. The column effluent was split into two using a “T junction” placed after the chromatographic column and analyzed “on-line” both by UV–Vis and heated electrospray ionization mass spectrometry (HESI/MS); 80 % of the effluent was sent to the Diode Array Detector (wavelength range 200–800 nm) while 20 % of the effluent was analyzed by HESI/MS. Mass spectra were recorded from *m/z* 100 to 2000 in negative and positive ionization modes. The capillary voltage was set at −27 V, the spray voltage was at 4 kV and the tube lens offset was at −87 V in the first case while, in positive ion mode, the capillary voltage was set at 4 V, the spray voltage was at 4 kV and the tube lens offset was at 140 V. The heater temperature was set at 200 °C and the capillary temperature was 275 °C. Data were acquired in MS and MS^n^ scanning mode. Data-dependent MS^n^ scanning was performed to minimize the total analysis time as it can trigger the fragmentation spectra of the target ions. Nitrogen was used as the sheath and the auxiliary gas. Helium served as the collision gas. The isolation width was 2 amu, and the normalized collision energy was 35 % for all compounds. Collision-induced dissociation (CID) was conducted in LTQ with an activation q of 0.25 and activation time of 30 ms. Zoom scan analyses were carried out to determine the charge state of molecular ions originating from some of the ellagitannin-based compounds.

##### 2.2.2.5 HPLC-UV-Vis analyses

Quantitative HPLC analyses of the main phenolic compounds identified in extracts from pomegranate samples were conducted on an HP 1100 Series HPLC (Agilent Technologies, Santa Clara, CA, USA) equipped with a binary pump and a UV–Vis detector. Individual phenols were separated on an XBridge BEH C18 column (130 Å, 5 mm, 4.6 mm × 150 mm, Waters) at a flow rate of 1 mL min^−1^; solvent A was water-acetonitrile-formic acid (95:4:1 v/v/v) and solvent B was water-acetonitrile-formic acid (44:55:1 v/v/v). The HPLC gradient elution was described in paragraph 2.2.2.4. Compounds were detected at 280 nm and 520 nm (anthocyanins). Individual phenolic compounds were quantified using calibration curves of the respective reference compounds. In particular, standard curves for ellagic acid, gallic acid, vanillic acid, caffeic acid, and ferulic acid were prepared over a concentration range of 1–50 μg mL^−1^ with five different concentration levels and duplicated injections at each level. The peak area of each polyphenol standard was calculated and plotted against the corresponding concentration using weighted linear regression to generate standard curves. Ellagitannins were quantified as ellagic acid equivalents, whereas gallotannins were as gallic acid equivalents. Anthocyanins quantification was performed with external calibration curves generated by repeated injections of a fixed volume of standard solutions of cyanidin-3-*O*-glucoside and delphinidin-3-*O*-glucoside over a concentration range of 0.1–50 μg mL^−1^ with five different concentration levels and duplicated injections at each level. All samples were prepared and analyzed in duplicate. The results were expressed as mg 100 g^−1^ fw.

### 2.3 Statistical analysis

Qualitative and biochemical parameters were statistically analyzed performing for each experiment a multifactor ANOVA (for P ≤ 0.05), to evaluate the effect of treatments (CW-CF, RW-SF, RW-CF), storage time (0 and 45 d), and their interaction. Statistica software (version 6.0, StatSoft, Inc., Tulsa, OK, USA) was used for this statistical analysis. In addition, parameters affected by factors were subjected to the Student-Newman-Keuls (SNK) test to evaluate significant differences among means. In the case of significant interactions, data were represented as histograms.

## 3 Results and discussion

### 3.1 Conventional and reclaimed water quality

During the first and second irrigation seasons (2020 and 2021 respectively), about 740 and 1100 m^3^ ha^−1^ (1111 and 1651 L per tree) of water for each treatment were applied between May and September, with the last irrigation turn carried out two weeks before the harvest. The irrigation started the third week of May in 2020 and the first week of June in 2021, and the water consumption was monitored by water meters installed for each irrigation water source. In each irrigation season, 9 water sample analyses (about one every 15 d) were conducted to monitor the principal chemical parameters reported in Table 2.

**Table 2:** Value of pH, electrical conductivity water (EC), and inorganic solute content anions and cations measured in conventional water (CW) and reclaimed water (RW) collected during the first (2020) and second (2021) seasons. Each data represents the mean of 18 values (± the standard deviation measured).

Treatments	2020		2021	
	CW	RW	CW	RW
pH	7.90 ± 0.25	7.41 ± 0.43	7.29 ± 0.34	6.83 ± 0.41
EC (μS/cm)	547 ± 12.5	2154 ± 109	503.2 ± 66.4	1563 ± 259
**Parameters (mg L**^**−**^**^1^)**				
B^3+^	0.10 ± 0.02	0.11 ± 0.04	0.11 ± 0.01	0.13 ± 0.03
Ca^2+^	39.3 ± 1.15	91.7 ± 5.15	33.4 ± 5.76	75.3 ± 6.55
K+	9.10 ± 1.00	34.1 ± 1.65	10.4 ± 1.34	25.1 ± 4.93
Mg^2+^	17.0 ± 1.00	39.5 ± 2.39	14.1 ± 1.74	25.1 ± 4.45
Na^+^	46.7 ± 2.51	234.1 ± 10.13	47.7 ± 5.44	188 ± 38.6
F^−^	0.25 ± 0.07	0.31 ± 0.05	0.35 ± 0.04	0.26 ± 0.03
Cl^−^	49.3 ± 1.15	311.1 ± 42.1	47.48 ± 5.15	282 ± 59.5
Br^−^	0.09 ± 0.01	0.64 ± 0.19	0.10 ± 0.01	0.87 ± 0.22
NO_3_^−^	2.33 ± 0.57	32.4 ± 15.2	1.66 ± 0.42	43.4 ± 43.1
NH_4_^+^	<0.1	30.3 ± 18.1	<0.1	37.1 ± 12.1
Total P	<0.1	5.08 ± 1.14	<0.1	2.57 ± 1.31
SO_4_^2-^	41.3 ± 5.31	291.1 ± 78.1	59.9 ± 6.82	336 ± 95.1

The irrigation water's characteristics indicated that the pH values for CW and RW were neutral, while many other parameters for RW were higher than in CW. For example, RW was 3 times more saline than CW, and at the same time, most other nutrients were significantly higher in RW. In addition, all the parameters did not exceed the international standards and were still below the limits, except for Cl^−^ which was slightly above the limit [41].

This excess is not significant even though, no damage was reported or noticed during the experiment. Moreover, the available NO_3_^−^ is highly benefic for the plants, since it is the most preferable N form for plants as reported in the literature [14]. As pomegranate trees are moderately tolerant to salinity, RW can be exploited for irrigation due to its constituents of beneficial nutrients, which can serve as a sustainable strategy in saving chemical fertilizers costs, protecting the environment, and feeding pomegranate trees continuously [42,43]. Similar results have been reported in the literature, where smart fertigation saved up to 50 % of water, and the cost of fertilizers and pesticides was lower than that of freshwater and chemical fertilizers [18,43]. In general, the nutrients present in the RW are considered bioavailable and sustainable. On the contrary, nutrients from chemical fertilizers are available immediately but unsustainable. This difference in sustainability can result in nutrient loss with drainage for chemical fertilizers. The presence of all essential elements for pomegranates can be noticed in the RW. In addition, other micronutrients are not shown, but they are still present in adequate quantity and below limits. All the trees irrigated with RW showed normal growth, and no essential-element deficiency symptoms were reported during the growing stage (field observation).

### 3.2 Dimension and weight of pomegranate fruit

At harvest time, the pomegranates divided into three weight classes showed a more elevated % of fruit (over 40 %), in the range of 400–500 g for the harvest carried out in the second year in CW-CF and RW-CF treatments. On the contrary, the treatment with the highest percentage of fruit in the range 200–300 g was shown by RW-SF; in particular, in this treatment, few differences among fruit class and harvest year were measured (Table 3), and the distribution of weight was balanced between the groups.

**Table 3:** Percentage of the different pomegranate fruit weight classes, measured for each irrigation treatment (CW-CF: conventional water + conventional fertigation; RW-CF: reclaimed water + conventional fertigation; RW-SF: reclaimed water + smart fertigation) during seasons 2020 and 2021.

Average fruit weight	CW-CF		RW-CF		RW-SF	
	2020	2021	2020	2021	2020	2021
(g)	**%**					
500–400	38.9	43.3	35.0	42.6	34.9	37.0
400–300	37.1	38.5	33.9	37.5	34.3	32.4
300–200	24.0	18.2	31.1	19.9	30.8	30.6

**Fig. 1:**
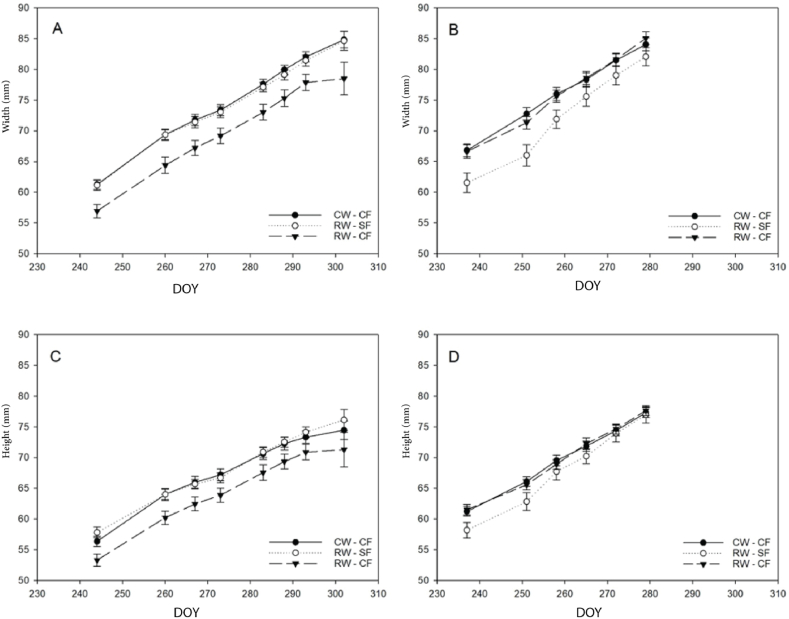
Width (A, B) and height (C–D) of pomegranates fruit during day of year (DOY) in 2020 (A–C) and (B–D) 2021 seasons, using different irrigation treatments (CW-CF: conventional water + conventional fertigation; RW-CF: reclaimed water + conventional fertigation; RW-SF: reclaimed water + smart fertigation).

Regarding the dimensions of the pomegranate, in the first season (2020), the highest value of fruit's width was reported for CW-CF and RW-SF treatments, whereas the lowest one was for RW-CF during all sampling times. In addition, during the second season, few differences were recorded for the width of fruit among the treatments with the same value at harvest time on average. As regards the length, during the first year, the fruit treated with RW-CF showed the lowest values compared to RW-SF and CW-CF treatments, the latter reporting the highest length fruit value. No differences among treatments were observed during the second season (2021) at harvest time (Fig. 1). All the width and length of pomegranate fruit are within the normal range of this variety. *Punica granatum* L. cv Wonderful One typically ranges from 6 to 12 cm, which confirms the effectiveness and potential of using RW for irrigation.

**Fig. 2:**
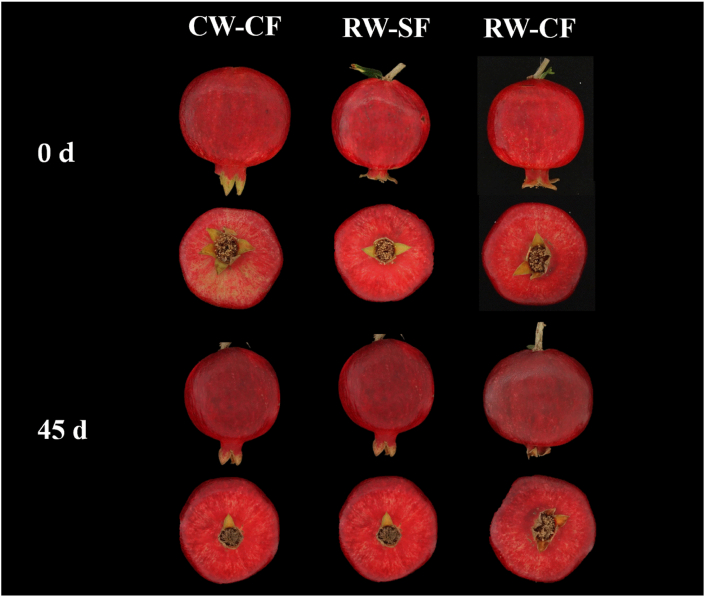
Fruit of pomegranates at harvest (0 d) and after 45 d of the storage at 7 °C applying different irrigation treatments (CW-CF: conventional water + conventional fertigation; RW-CF: reclaimed water + conventional fertigation; RW-SF: reclaimed water + smart fertigation).

About the value of aspect ratio (data not shown) of the fruit, few differences were observed among CW-CF, RW-CF, and RW-SF with 1.10 (±0.06), 1.10 (±0.06), and 1.13 (±0.05), respectively during the first season. On the contrary, in the second year, the mean value for RW-SF was 1.07 (±0.05), whereas 1.09 (±0.05) for the CW-CF and 1.10 (±0.05) for RW-CF treatment. The regular dimension of fruit is a very important parameter in sorting. It affects the size of the box packing and determines the number of fruits that can be placed in each shipping container or plastic bag [44]. As mentioned above, the fruit irrigated with RW-SF have balanced weight classes, which can be beneficial for the commercial process. This is in addition to the important water and nutrient-saving quantities when using RW, which shows sustainability in different aspects (economic, environmental, and social). The combination of RW-SF seems to be a promising strategy to reduce the pressure on freshwater resources and the environment (fewer mineral fertilizers), especially in the Apulia region, where water is scarce due to the extensive agricultural production and climate change impacts.

### 3.3 Changes in quality parameters, at harvest and after storage of pomegranates fruit

Pomegranates fruit grown under the two different systems of irrigation (CW and RW) and fertigation (CF and SF), showed similar values of the main quality parameters analyzed: pH, TSS, TA, and MI. In both years, these remained unchanged throughout the storage period (Table 4).

**Table 4:** Effect of irrigation treatment (CW-CF: conventional water + conventional fertigation; RW-SF: reclaimed water + smart fertigation; RW-CF: reclaimed water + conventional fertigation) and storage days (0 d and after 45 d) on pH, total soluble solid (TSS), titratable acidity (TA) and maturity index (MI) of pomegranates fruit at harvest (0 d) and after 45 d of storage at 7 °C during seasons 2020 and 2021.

	2020						2021							
	pH	TSS	TA		MI		pH		TSS		TA		MI	
		° Brix	(%)						° Brix		(%)			
Treatments (A)														
CW-CF	2.9	16.4	3.10	a	5.3	b	3.4	b	16.0	b	3.26	a	4.9	b
RW-SF	2.9	16.5	2.80	b	5.9	a	3.5	a	16.7	a	2.95	a	5.7	a
RW-CF	2.9	16.8	2.80	b	6.0	a	3.5	ab	16.4	a	2.74	b	6.0	a
	ns	ns	*		*		*		***		**f**		**	
Day (B)														
0	2.9	16.4	3.40	a	4.8	b	3.3	b	16.5	a	3.11	a	5.3	
45	2.9	16.4	2.60	b	6.3	a	3.6	a	16.2	b	2.86	b	5.7	
	ns	ns	****		***		***		***		**		ns	
A X B	ns	ns	ns		ns		ns		***		ns		ns	

For each parameter and factor (treatments or days), means followed by different letters are significantly different according to Student–Newman–Keuls (SNK) test.

^(1)^ ns: not significant; **** significant for P ≤ 0.0001; *** significant for p ≤ 0.001; ** significant for p ≤ 0.01; * significant for p ≤ 0.05.

Regarding the color parameters, irrigation treatments, in both years, did not affect the fruit and juice color (Table S1). Similarly, pomegranate fruit coming from different irrigation treatments, do not show difference in visual quality as reported in Fig. 2.

Similar values were also detected by other authors on the same pomegranate cultivar [18,45]. On the contrary, data are in contrast with changes during the postharvest storage of pomegranate in which the increase in pH can be related to the use of organic acids which are the main contributors to TA, which is following the reduction of TSS content after the third month of storage [46,47]. The results, reported in Table 4, showed that RW, especially with SF, did not negatively impact the photosynthesis process, and, therefore, all the parameters of fruit are within the range of good quality.

In addition, previous studies on nectarines demonstrated that fruit quality parameters such as TSS, TA, and pH were affected by the type of water used in the experiment [48]. During three irrigations seasons, the nectarines fruit quality parameters, were improved when irrigating with RW. The positive effect of nutrients and salinity in RW can be noticed obviously, and it can also be explained by the fact that trees can adapt to salinity stress by increasing the biosynthesis of soluble solids, sugars, proteins, amino acids, and other secondary metabolites. The presence of these compounds can enhance the nutritive value and the fruit quality. Other studies, showed similar trends for different crops (tomatoes, nectarine, almonds, etc.), but also the values obtained in our study on RW-SF and RW-CF compared to CW can suggest an early ripening on pomegranates fruit [10,11,14]. In particular, the fruit of trees irrigated with RW, showed less TA in both years and higher TSS only during the second season (2021), confirming that RW stimulate early ripening. In general, more long-term studies are still missing to evaluate the impact of RW on pomegranate trees, but these results suggest a good potential of RW in preserving a good quality of fruit while saving water and fertilizers.

**Fig. 3:**
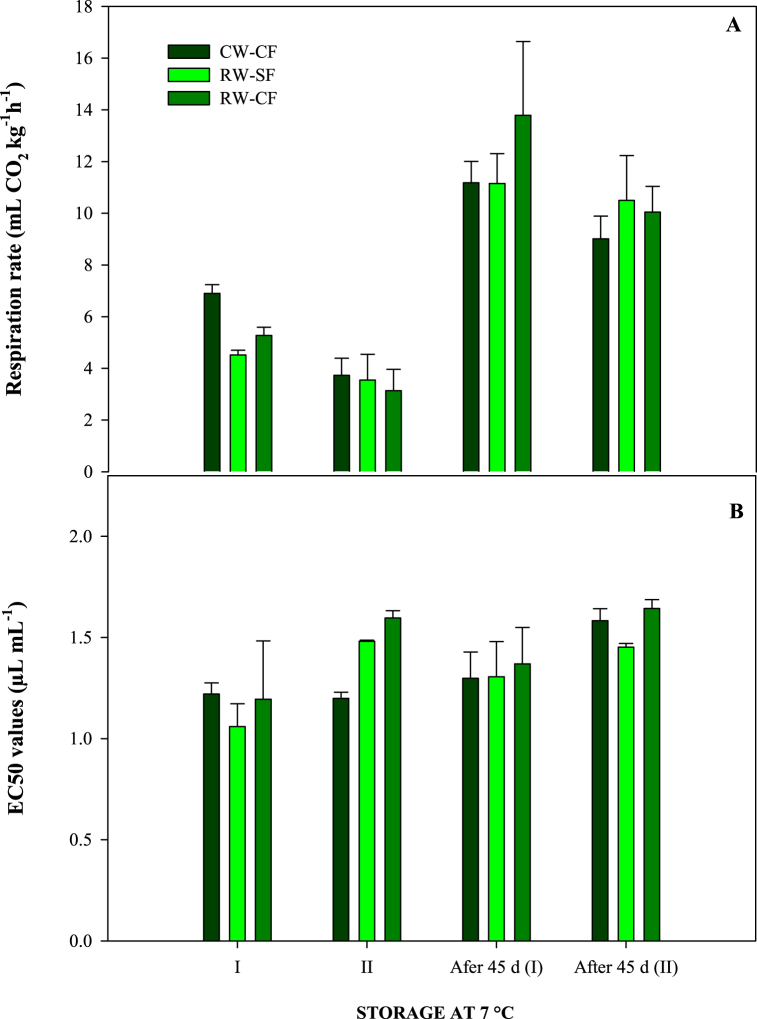
Respiration rate (A) and EC50 value (B) measured at 0 d and 45 d of pomegranate fruit coming from different irrigation treatments (CW-CF: conventional water + conventional fertigation; RW-CF: reclaimed water + conventional fertigation; RW-SF: reclaimed water + smart fertigation) during seasons 2020 (I) and 2021 (II).

In Fig. 3, the respiration rate and the antioxidant activity of pomegranates are reported. For the respiration rate, at harvest, very low values were detected (mean values around 6 and 3-mL CO_2_ kg^−1^ h^−1^ for the first and the second year, respectively), then after 45 days of storage, about a 2-fold increase was measured in both years (Fig. 3A). According to Ref. [19], pomegranate is classified as a non-climacteric fruit due to its low respiration and ethylene production rates after harvest. Generally, very low differences were noticed among treatments, probably due to the variability among samples. The values recorded at harvest, agree with previous research papers on pomegranates (cv Wonderful) [19,49], while slight increase showed at the end of the storage is probably due to the incoming senescence of the product. This result demonstrated that any treatments affect physiological injuries to pomegranate fruit during storage.

The EC_50_ of pomegranate juice was between 1 and 1.6 μL mL^−1^, when measured at harvest in two consecutive years, and similar values were detected after 45 d of storage (Fig. 3B). The small differences between treatments are probably due to the variability among samples. At harvest, similar values of EC_50_ (1.97 ± 0.27 μL mL^−1^) were also measured by Ref. [50] on cv Wonderful. However, the results showed that fruit irrigated with RW applying conventional or smart fertigation preserved the antioxidant activity also during storage. Based on these interesting results, a deep biochemical characterization was carried out during the second year, and the results are reported in the next paragraphs.

### 3.4 Identification and quantification of polyphenols at harvest and after postharvest storage of pomegranates fruit

Separation, identification, and quantification of phenolic compounds contained in fresh and stored pomegranate fruit coming from the second year were carried out by UHPLC-MS^n^ and HPLC-UV-Vis analyses.

**Fig. 4:**
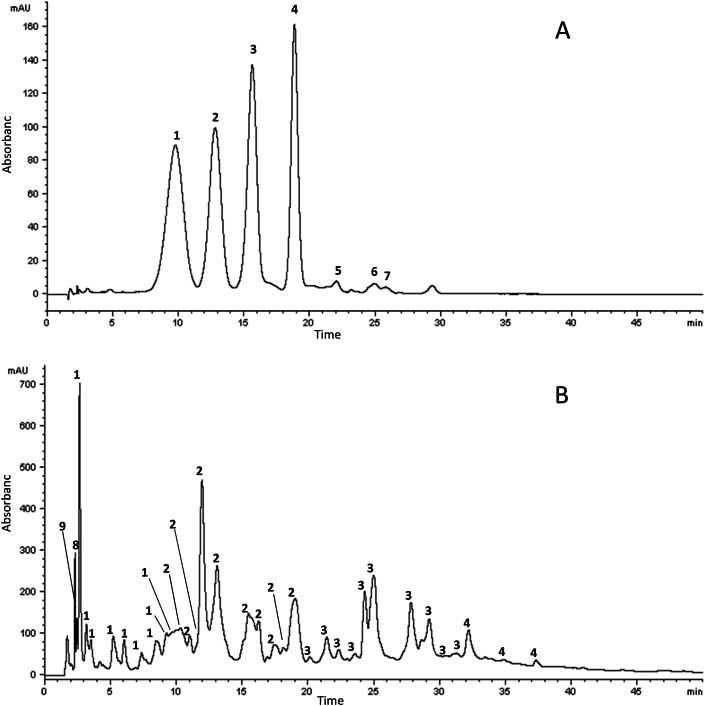
HPLC-UV-Vis chromatogram recorded at 520 nm (A) and 280 nm (B) of phenolic compounds present in freshly pomegranate fruit (0 d)*,* grown using conventional water + conventional fertigation (CW-CF). For peak assignments, see Table S2.

The overall analysis led to the detection and tentatively identification of 52 compounds based on the interpretation of their fragmentation patterns obtained from mass spectra (MS^n^ experiments) and by comparison with the literature. In addition, 5 compounds were detected but not identified. The classes of phenolic compounds detected in these analyses agreed with those already reported in previous studies on phenolic profile of pomegranate arils and juice [[51], [52], [53], [54], [55], [56]] and included anthocyanins, hydrolyzable tannins, hydroxycinnamic acids, vanillic acid derivatives, organic acids and a neolignan. In particular, the HPLC-UV-Vis chromatogram of phenolic compounds detected at harvest in pomegranate samples grown using CW-CF is shown in Fig. 4; while the list of compounds identified in this sample is reported in Table S2. As to anthocyanins, fresh pomegranate contained delphinidin 3,5-diglucoside (peak 1), delphinidin 3-glucoside (peak 3), cyanidin 3,5-diglucoside (peak 2), cyanidin 3-glucoside (peak 4), cyanidin 3-pentoside (peaks 6 and 7) and pelargonidin 3-glucoside (peak 5) which were known to be present in pomegranate arils and juice [[55], [56], [57]]. The results are reported below (Table S2, Fig. 4 A).

Hydrolysable tannins, both gallotannins and ellagitannins, were the most represented class of phenolic compounds, with many molecules belonging to ellagitannins, and many isomeric forms were detected.

The identification of some ellagitannins-based compounds was also achieved by using the high-resolution “zoom scan” analyses, a technique that improves resolution of the ^12^C/^13^C isotope pattern of their molecular ions, thus allowing the assessment of charge state and, consequently, the determination of the correct molecular weight [59] For example, the mass spectrum of peak 21 (Fig. 4B) had [M − H]^-^ ions at *m*/*z* 1417 and 708, which was shown to be doubly charged by zoom-scan analysis, confirming the molecular weight of this compound (1418 Da). Based on the comparison of the MS^2^ spectra with mass spectral data reported by Ref. [58]. This peak was tentatively identified as camptothin A, a dimeric hydrolyzable tannin isolated from the leaf of *Camptotheca acuminate* by Ref. [60]. To the best of our knowledge, this is the first report describing the presence of camptothin A in pomegranate arils.

Furthermore, an abundant ion at *m*/*z* 783, showed to be doubly charged by zoom scan, and a less abundant ion at *m*/*z* 1567 were present in the mass spectra of peaks 23, 27 and 28 (Fig. 4B). Based on the MS^n^ spectra, these peaks were tentatively identified as oenothein B and its isomers. Oenothein B is a dimeric hydrolysable tannin isolated from the leaves of *Oenothera erythrosepala* Borbás by Ref. [60]and found in pomegranate arils [54]. Moreover, it is known that oenothein B has two isomers, oenothein D and F, isolated from roots and stems of *Oenothera laciniata* by Ref. [61]. Furthermore, an ion at *m*/*z* 1085 is also present in the mass spectra of peaks 27 and 28 (Fig. 4B) that, based on the MS^2^ spectra, was identified as tri-(hexahydroxydiphenoyl (HHDP))-hexoside. Peaks 34 and 35 (Fig. 4B) exhibited an ion at *m*/*z* 784, corresponding to the pseudo molecular doubly charged ion [M − H]^−2^, as established by zoom scan analysis, and a less abundant ion at *m*/*z* 1569. According to the mass spectral data, peaks 34 and 35 were identified as eucalbanin B and its isomer. As a matter of fact [62], isolated from the fruit extract of *Eucalyptus alba* Reinw. an isomer of eucalbanin B, defined as *Eucalbanin* C. *Eucalbanin* B was previously detected in pomegranate arils by Ref. [54]. Peaks 36, 37 and 39 (Fig. 4B) presented two ions at *m*/*z* 1176 and *m*/*z* 784 that corresponded to the pseudo-molecular doubly and triply charged ions, respectively, as established by zoom scan analysis. In addition, the mass spectral data showed that this compound was identified as the trimer eucarpanin T1, previously detected in arils of *Punica granatum* L. by Ref. [54] and, probably, its two isomers. In the mass spectra of peak 36 is also present an ion at *m*/*z* 951 that, in the MS^2^ experiment, produced fragment ions at *m*/*z* 933, and 915, due to the losses of one and two water molecules, respectively. This compound was tentatively identified as galloyl-HHDP-dehydrohexahydroxydiphenoyl (DHHDP)-hexoside (granatin B) based on the mass spectral data reported in a previous study [51]. The peak 20 (Fig. 4B) also showed the same [M − H]^-^ ion at *m*/*z* 951. This compound had major fragment ions at *m*/*z* 907 ([M − H - CO_2_]^-^), *m*/*z* 783 in the MS^2^ spectrum and further fragment ions in the MS^n^ spectra at *m*/*z* 605 [M − H - CO^2^ - HHDP]^-^, *m*/*z* 481 (HHDP-hexoside) and *m*/*z* 301 and was tentatively identified as HHDP-valoneoyl-glucoside. In the mass spectrum of peak 36 (Fig. 4B), an ion at *m*/*z* 953 was also present, identified as galloyl-chebuloyl-HHDP-glucose (chebulagic acid) based on the mass spectral data, which has previously been detected in the fruit of *Terminalia* species [63]. To the best of our knowledge, chebulagic acid is here detected in pomegranate arils for the first time.

In the HPLC peaks 12, 15, and 29 (Fig. 4B) compounds with the same pseudomolecular ion ([M − H]^-^) at *m*/*z* 643 were present but had slight differences in the fragmentation pattern. MS^n^ analyses of these compounds did not lead to a definitive identification. These compounds had already been found in pomegranate juice and generically referred to as ellagitannins by others, but remained unidentified [53,55,64].

HPLC/ESI-ITMS^n^ analyses of the fresh pomegranate arils extract showed a compound with pseudomolecular ion [M − H]^-^ at *m*/*z* 555 in peak 31 (Fig. 4B). In the MS^2^ spectrum, the following fragmentation pattern was obtained: *m*/*z* 537, due to the loss of a water molecule, *m*/*z* 393 corresponding to the deprotonated ion after the loss of a hexose moiety [M − H - 162], and *m*/*z* 197. The *m*/*z* 197 ion was further subjected to MS^3^ analysis showing signals at *m*/*z* 182 and 153 that are peculiar ions of syringic acid. Therefore, the compound eluted in peak 31 was tentatively identified as syringic acid derivative. A second compound eluted at the same retention time (peak 31, Fig. 3B) was identified as ellagic acid hexoside. The compound present in peak 32 (Fig. 4B) displayed pseudo molecular ion at *m*/*z* 551 and fragment ions at *m*/*z* 491, 461, 431 and 389 (loss of 60, 90, 120, and 162 Da), suggesting the presence of a C-hexosyl group. Furthermore, MS^3^ scan of the ion at *m*/*z* 389 produced the specific fragmentation pattern of the ferulic acid. Therefore, the compound eluted in peak 32 was tentatively identified as a ferulic acid-*C*-hexoside derivative.

Only one lignan was identified in fresh pomegranate arils. In particular, peak 40 showed a pseudomolecular ion ([M − H]^-^) at *m*/*z* 507 in the mass spectrum and two fragment ions at *m*/*z* 327 and 345 in the MS^2^ spectrum, originating from the neutral loss of the glucose moiety (Fig. 4B). This compound was identified as pomegralignan, a glucose ester of neolignan found for the first time in arils of *Punica granatum* L. by Ref. [54] and observed in pomegranate juice [65].

Individual phenolic contents measured by HPLC-UV/Vis in fresh pomegranate fruit grown using CW-CF are reported in Table S3. Ellagitannins were the most predominant class of phenolic compounds, accounting for about 73 % of the total phenolic content in the fresh sample, followed by anthocyanins which constituted 11.39 % of the total phenolic content.

**Fig. 5:**
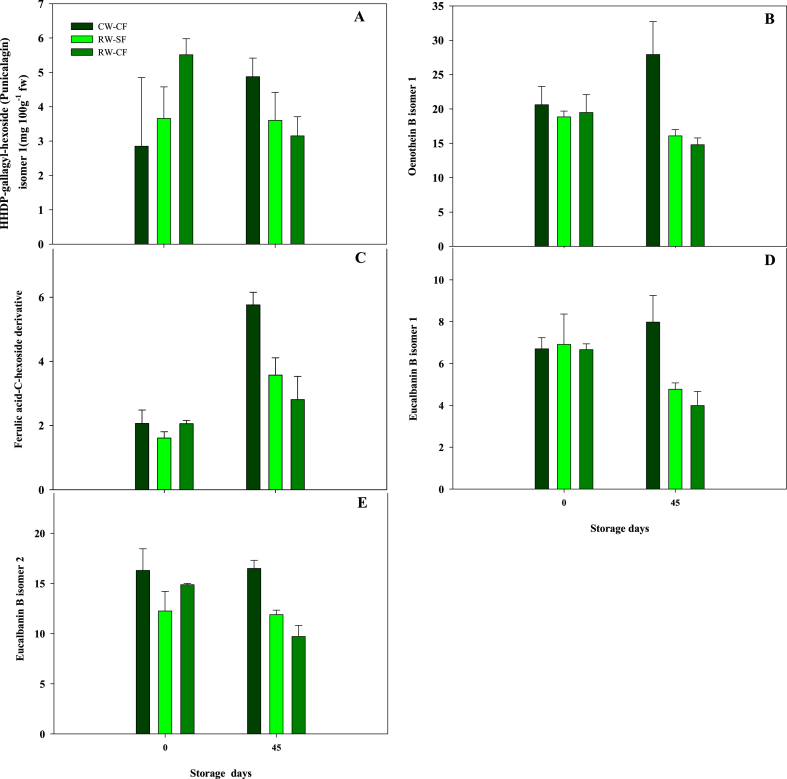
Main effect of the interaction of treatments (CW-CF: conventional water + conventional fertigation; RW-CF: reclaimed water + conventional fertigation; RW-SF: reclaimed water + smart fertigation) and storage time (0 and 45 d at 7 °C) on phenolic compounds identified in pomegranates fruit.

Results of the multifactor ANOVA performed on data obtained by quantitative analysis of total and individual polyphenols in extracts of pomegranates fruit, grown using different irrigation water sources and fertigation treatments, at harvest and after storage are reported in Fig. 5 and Table 5.

**Table 5:** Main effect of irrigation treatments (CW-CF: conventional water + conventional fertigation; RW-CF: reclaimed water + conventional fertigation; RW-SF: reclaimed water + smart fertigation) and storage time (0 and 45 days at 7 °C) on phenolic compounds identified in pomegranates fruit.

Phenolic compounds		Treatment						Phenolic compounds		Storage time			
		CW-CF		RW-SF		RW-CF				0 d		45 d	
		(mg 100g^−1^ fw)								(mg 100g^−1^ fw)			
1	Delphinidin 3,5-diglucoside	7.50	a	5.25	b	3.96	b	5	Pelargonidin 3-glucoside	0.23	b	0.36	a
2	Cyanidin 3,5-diglucoside	4.50	a	2.68	b	3.3	b	10	Galloyl hexoside	6.78	a	5.72	b
20	HHDP-valoneoyl-glucoside	8.98	a	4.17	b	3.56	b	13	Galloyl-HHDP-gluconic acid (Lagerstannin C) isomer 2	3.75	a	2.25	b
21	Ellagitannin (Camptothin A)*	3.85	a	2.86	b	2.48	b	18	Caffeic acid hexoside	4.01	a	0.00	b
23	Oenothein B isomer 1	24.27	a	17.47	b	17.14	b	30	Caffeic acid hexoside isomer	1.70	a	0.00	b
24	Digalloyl-HHDP-hexoside isomer (Pedunculagin II) isomer 1 + HHDP-gallagyl-hexoside (Punicalagin) isomer 2	19.67	a	16.67	b	15.32	b	37	Eucarpanin T1 isomer 2 + Ellagic acid-deoxyhexoside + Ellagic acid-pentoside	9.12	a	6.53	b
27	Tri-HHDP-hexoside + Oenothein B isomer 2	5.05	a	4.58	b	3.85	b	38	Ellagic acid	2.40	b	4.50	a
								39	Eucarpanin T1 isomer 3	3.50	a	2.30	b
								42	Guaiacyl (8–5) ferulic acid hexoside	1.00	a	0.80	b
													
		CW-CF		RW-SF		RW-CF				0 d		45 d	
		(mg_GAE_ g^−1^ fw)								(mg_GAE_ g^−1^ fw)			
	Total Phenolic Contents	2.90	a	2.71	a	2.17	b		Total Phenolic Contents	2.39	b	2.79	a
													

Different lowercase letters indicate statistical differences within treatment and storage time according to LSD test (P ≤ 0.05).

^⁎^ Compound 21, tentatively identified based on the mass spectral data cited by [58]

Abbreviations used: HHDP, hexahydroxydiphenoyl; DHHDP, dehydrohexahydroxydiphenoyl.

These results showed that total polyphenols content, delphinidin 3,5-diglucoside, cyanidin 3,5-diglucoside, HHDP-valoneoyl-glucoside and ellagitannin (camptothin A) 1 were significantly negatively affected by treatments. In addition, the treatments also changed the area of the HPLC peak containing Pedunculagin II isomer 1 and Punicalagin isomer 2, and that of the peak containing tri-HHDP-hexoside and Oenothein B isomer 2, thus suggesting that the amount of either one or both the molecules present in those peaks was affected by the treatment (CW-CF, RW-CF and RW-SF) (Table 5).

On the other hand, pelargonidin 3-glucoside, galloyl hexoside, lagerstannin C isomer 2, caffeic acid hexoside, caffeic acid hexoside isomer, ellagic acid, eucarpanin T1 isomer 3 and guaiacyl(8–5)ferulic acid hexoside, were significantly affected only by storage time. Caffeic acid hexoside and caffeic acid hexoside isomer, present in fresh pomegranates, were not found in pomegranate samples after 45 d of storage. In addition, the storage time also changed the area of the HPLC peak containing eucarpanin T1 isomer 2, ellagic acid-deoxyhexoside and ellagic acid-pentoside, thus suggesting that the amount of these molecules was affected by the storage time, as well as the total polyphenols content. Instead, the amount of punicalagin isomer 1, ferulic acid-*C*-hexoside derivative, eucalbanin B isomer 1 and eucalbanin B isomer 2 was affected by the interaction of both parameters (storage time and treatment). Moreover, oenothein B isomer 1 content was affected by treatment and the interaction of both parameters (storage time and treatment) (Table 5 and Fig. 5). The other compounds' content was not affected by these factors.

In general, the use of RW showed good performance and a positive impact on fruit quality parameters. The results are promising for future considerations and implementation of the RW as a strategy to mitigate the impact of water scarcity on our societies and agroecosystems. In terms of dimensions of the pomegranate fruits, no significant differences were noticed, while for the fruit quality parameters (TSS and TA) and total phenolic compounds, RW-SF showed good results like the CW-CF treatment, even if there was an impact of treatments on some specific phenolic compounds after 45 days of storage. RW-SF treatment showed balanced and optimal results in terms of average fruit weight during the two seasons Regarding the phenolic composition, pomegranate fruits contained mainly hydrolyzable tannins and anthocyanins and, generally, when comparing treatments, the identified compounds showed a very small reduction (about 17 %) in fruit. irrigated with RW (in both fertigation conditions) to control. For total phenolic contents, no significant difference between CW-CF and RW-SF (2.90 and 2.71 mg GAE g^−1^ fw, respectively) was noticed, while RW-CF had the lowest total phenolic contents (2.17 GAE g^−1^ fw), but some phenolic compounds were affected by treatments and by storage time as described above. The bioavailability of nutrients in RW with smart fertigation positively impacted the fruit quality, resulting in normal tree growth without any damage or deficiency symptoms. Moreover, the pomegranate fruits irrigated with RW had a typical size of the variety used in this study (*Punica granatum* L. cv Wonderful One), which also confirmed RW's effectiveness as a promising irrigation strategy. These results align with previous studies on crops irrigated with RW under various treatments and conditions, all of which showed positive outcomes in terms of yield and fruit quality parameters.

## 4 Conclusion

Pomegranate trees (*Punica granatum* L. cv Wonderful One) were irrigated, during two consecutively seasons, using reclaimed or conventional water applying conventional or smart fertigation systems. The results showed that reclaimed water stimulated early ripening in the fruit, preserving the antioxidant activity, the total phenol content and the content in hydrolyzable tannins and anthocyanins. Moreover, the use of reclaimed water allowed to maintain the fruit quality and not cause physiological injuries to pomegranate fruit during storage.

However, it's important to conduct extensive, long-term studies to assess the sustainability and effectiveness of using reclaimed water in different environmental conditions and for various fruit tree crops. In conclusion, the results of these studies will provide valuable insights into the scalability, sustainability, and practicality of using reclaimed water in agriculture. This, in turn, can lead to the development of more efficient and sustainable water management practices, aligning with the goals of the current European Green Deal roadmap and the circular economy for sustainable future agriculture.

## Data availability

Data will be made available on request.

## Funding

This study was funded by the 10.13039/501100009886Regione Puglia as the project “Sistema innovativo di monitoraggio e trattamento delle acque reflue per il miglioramento della compatibilita ambientale ai fini di un'agricoltura sostenibile” -SMART WATER (grant no. 5ABY6P0) through the INNONETWORK CALL 2017.This study was partially carried out within the Agritech National Research Center and received funding from the European Union Next-Generation EU (Piano Nazionale di Ripresa e Resilienza (Pnrr) – Missione 4 Componente 2, Investimento 1.4 – D.D. 1032 17/06/2022, CN00000022) and Project “SUS&LOW­ Sustaining low­ impact practices in horti­culture through non-destructive approach to provide more information on fresh produce history and quali­ty” (grant number: 201785Z5H9) Prin 2017 from the 10.13039/501100003407Italian Ministry of Education University. This manuscript reflects only the authors' views and opinions, neither the EU nor the 10.13039/501100000780European Commission can be considered responsible for them. Part of this study was conducted using facilities provided by PON R&I 2014–2020 PIR01_00017 NRbiomics Project.

## Ethics statement

N/A.

## Author agreement statement

The Corresponding Author declares that this manuscript is original, has not been published before and is not currently being considered for publication elsewhere. The Corresponding Author confirms that the manuscript has been read and approved by all named authors and that there are no other persons who satisfied the criteria for authorship but are not listed. The Corresponding Author further confirms that the order of authors listed in the manuscript has been approved by all of us. The Corresponding Author is the sole contact for the Editorial process. He is responsible for communicating with the other authors about progress, submissions of revisions, and final approval of proofs.

## CRediT authorship contribution statement

**Michela Palumbo:** Writing – original draft, Formal analysis, Data curation. **Virginia Carbone:** Writing – original draft, Supervision, Formal analysis, Data curation. **Ilde Ricci:** Formal analysis, Data curation. **Bernardo Pace:** Writing – review & editing, Writing – original draft, Supervision, Methodology, Formal analysis, Conceptualization. **Maria Cefola:** Writing – review & editing, Writing – original draft, Methodology, Formal analysis, Data curation, Conceptualization. **Paola Minasi:** Formal analysis, Data curation. **Simone Pietro Garofalo:** Data curation. **Salvatore Camposeo:** Supervision. **Anas Tallou:** Writing – original draft. **Gaetano Alessandro Vivaldi:** Writing – review & editing, Writing – original draft, Methodology, Investigation, Funding acquisition, Conceptualization.

## Declaration of competing interest

The authors declare that they have no known competing financial interests or personal relationships that could have appeared to influence the work reported in this paper.
