# Supplementary material for: Qualitative and biochemical characteristics of pomegranate fruit grown using reclaimed water and low input fertigation treatments at harvest and during storage
Source: Heliyon. 2024 Jul 14;10(14):e34430. doi: 10.1016/j.heliyon.2024.e34430 (PMC11315205; doi:10.1016/j.heliyon.2024.e34430)
Supplement: Multimedia component 1 [file mmc1.docx]

**Table S1.** Fruit and juice color parameters of pomegranate at harvest (0 d) and after storage (45 d) at 7 °C grown using different irrigation treatments (CW-CF: conventional water + conventional fertigation; RW-CF: reclaimed water + conventional fertigation; RW-SF: reclaimed water + smart fertigation) at seasons 2020 and 2021.

|  | 2020 |  | 0 d | | | | | | | | | | |  | 45 d | | | | | | | | | | | | | |
| --- | --- | --- | --- | --- | --- | --- | --- | --- | --- | --- | --- | --- | --- | --- | --- | --- | --- | --- | --- | --- | --- | --- | --- | --- | --- | --- | --- | --- |
|  | Color parameters | | CW-CF | | |  | RW-SF | | |  | RW-CF | | |  | CW-CF | | |  | RW-SF | | | |  | | RW-CF | | | |
| Skin | *L** |  | 39.80 | ± | 1.16 |  | 41.88 | ± | 0.49 |  | 39.16 | ± | 0.43 |  | 39.22 | ± | 1.76 |  | 40.31 | ± | 1.59 |  | | 38.60 | | ± | 1.98 |  |
|  | *a** |  | 40.68 | ± | 1.18 |  | 42.26 | ± | 0.27 |  | 40.57 | ± | 0.43 |  | 42.29 | ± | 1.07 |  | 42.23 | ± | 1.72 |  | | 39.67 | | ± | 1.08 |  |
|  | *b** |  | 19.09 | ± | 0.51 |  | 20.59 | ± | 0.69 |  | 19.58 | ± | 0.38 |  | 20.40 | ± | 2.25 |  | 20.48 | ± | 1.51 |  | | 18.11 | | ± | 1.79 |  |
|  | h° |  | 25.16 | ± | 1.22 |  | 25.98 | ± | 0.70 |  | 25.76 | ± | 0.57 |  | 25.69 | ± | 1.87 |  | 25.86 | ± | 1.34 |  | | 24.53 | | ± | 2.36 |  |
|  |  |  |  |  |  |  |  |  |  |  |  |  |  |  |  |  |  |  |  |  |  |  | |  | |  |  |  |
| Juice | *L** |  | 16.81 | ± | 0.19 |  | 17.35 | ± | 0.45 |  | 16.83 | ± | 0.84 |  | 17.19 | ± | 0.97 |  | 16.25 | ± | 0.07 |  | | 16.60 | | ± | 0.43 |  |
|  | *a** |  | 16.49 | ± | 1.17 |  | 19.93 | ± | 0.80 |  | 19.90 | ± | 1.34 |  | 18.06 | ± | 2.06 |  | 16.46 | ± | 1.26 |  | | 16.67 | | ± | 0.87 |  |
|  | *b** |  | -9.62 | ± | 0.18 |  | -8.92 | ± | 0.55 |  | -9.00 | ± | 0.87 |  | -9.51 | ± | 0.54 |  | -9.87 | ± | 0.08 |  | | -9.58 | | ± | 0.15 |  |
|  | h° |  | -30.33 | ± | 2.17 |  | -24.13 | ± | 2.05 |  | -24.42 | ± | 3.46 |  | -27.97 | ± | 4.11 |  | -31.03 | ± | 2.15 |  | | -29.92 | | ± | 1.70 |  |
|  |  |  |  |  |  |  |  |  |  |  |  |  |  |  |  |  |  |  |  |  |  |  | |  | |  |  |  |
|  | 2021 |  | 0 d | | | | | | | | | | |  | 45 d | | | | | | | | | | | | | |
|  | Color parameters | | CW-CF | | |  | RW-SF | | |  | RW-CF | | |  | CW-CF | | |  | RW-SF | | | |  | | RW-CF | | | |
| Skin | *L** |  | 48.11 | ± | 0.66 |  | 45.12 | ± | 2.27 |  | 49.62 | ± | 2.34 |  | 45.84 | ± | 0.57 |  | 43.49 | ± | 0.65 |  | | 44.81 | | ± | 0.23 |  |
|  | *a** |  | 38.82 | ± | 3.60 |  | 39.55 | ± | 3.19 |  | 36.62 | ± | 4.41 |  | 44.00 | ± | 0.64 |  | 40.32 | ± | 1.34 |  | | 42.44 | | ± | 0.74 |  |
|  | *b** |  | 25.47 | ± | 0.35 |  | 21.99 | ± | 0.86 |  | 25.13 | ± | 0.35 |  | 27.59 | ± | 0.86 |  | 23.19 | ± | 1.25 |  | | 25.61 | | ± | 0.37 |  |
|  | h° |  | 33.37 | ± | 2.25 |  | 29.18 | ± | 3.01 |  | 34.63 | ± | 2.89 |  | 32.08 | ± | 1.04 |  | 29.90 | ± | 0.81 |  | | 31.11 | | ± | 0.13 |  |
|  |  |  |  |  |  |  |  |  |  |  |  |  |  |  |  |  |  |  |  |  |  |  | |  | |  |  |  |
| Juice | *L** |  | 13.52 | ± | 0.48 |  | 15.44 | ± | 3.67 |  | 19.00 | ± | 4.87 |  | 17.654 | ± | 2.5 |  | 19.119 | ± | 2.6 |  | | 17.83 | | ± | 0.8 |  |
|  | *a** |  | 12.13 | ± | 2.18 |  | 10.19 | ± | 3.35 |  | 8.61 | ± | 3.44 |  | 14.046 | ± | 0.9 |  | 18.892 | ± | 1.9 |  | | 17.05 | | ± | 0.9 |  |
|  | *b** |  | -12.96 | ± | 0.77 |  | -10.19 | ± | 3.78 |  | -9.81 | ± | 2.03 |  | -9.773 | ± | 1.2 |  | -8.482 | ± | 1.2 |  | | -9.007 | | ± | 0.6 |  |
|  | h° |  | -47.13 | ± | 6.86 |  | -44.60 | ± | 1.83 |  | -49.71 | ± | 5.03 |  | -34.83 | ± | 4.9 |  | -24.38 | ± | 5.2 |  | | -27.88 | | ± | 2.5 |  |

Data are mean of 3 values ± standard deviation.
