# Supplementary material for: Qualitative and biochemical characteristics of pomegranate fruit grown using reclaimed water and low input fertigation treatments at harvest and during storage
Source: Heliyon. 2024 Jul 14;10(14):e34430. doi: 10.1016/j.heliyon.2024.e34430 (PMC11315205; doi:10.1016/j.heliyon.2024.e34430)
Supplement: Multimedia component 2 [file mmc2.docx]

**Table S2**

List of phenolic compounds identified in pomegranate samples at harvest grown using conventional water + conventional fertigation (CW-CF) by UHPLC-MS^n^. Specific quasi-molecular ions and fragment ions are reported for each compound.

| **Peak** | **MS**  ***m/z*** | **MS^2^ ions**  ***m/z*** | **MS^3^ ions**  ***m/z*** | **MS^4^ ions**  ***m/z*** | **Proposed structure** |
| --- | --- | --- | --- | --- | --- |
| **1** | 627 [M]^+^ | 465, 303 | [627→303]: 285, 275, 257, 229, 201, 179, 153 |  | Delphinidin 3,5-diglucoside |
| **2** | 611 [M]^+^ | 449, 287 | [611→287]: 269, 241, 213, 189, 175, 161, 149, 137 |  | Cyanidin 3,5-diglucoside |
| **3** | 465 [M]^+^ | 303 | [465→303]: 285, 257, 229, 201, 173, 165 |  | Delphinidin 3-glucoside |
| **4** | 449 [M]^+^ | 287 | [449→287]: 269, 241, 213, 189, 185, 145, 137 |  | Cyanidin 3-glucoside |
| **5** | 433 [M]^+^ | 271 | [433→271]: 253, 225, 197, 173, 169, 145, 121 |  | Pelargonidin 3-glucoside |
| **6** | 419 [M]^+^ | 287 | [419→287]: 269, 241, 213, 189, 175, 149, 137 |  | Cyanidin 3-pentoside |
| **7** | 419 [M]^+^ | 287 | [419→287]: 269, 241, 213, 189, 175, 149, 137 |  | Cyanidin 3-pentoside |
| **Peak** | **MS**  ***m/z*** | **MS^2^ ions**  ***m/z*** | **MS^3^ ions**  ***m/z*** | **MS^4^ ions**  ***m/z*** | **Proposed structure** |
| **8** | 353 [M-H]^-^  391 [M-H]^-^ | 293, 191, 173, 155, 111  373, 217, 191 | [353→173]: 111  [353→111]: 67  [391→217]: 155, 111 | [353→173→111]: 67  [391→217→111]: 67 | Caffeoyl-isocitric acid  Citric acid derivative |
| **9** | 191 [M-H]^-^ | 173, 111 | [191→173]: 129, 111, 173 | [191→173→111]: 67 | Citric acid |
| **10** | 331 [M-H]^-^ | 271, 211, 193, 169, 125 | [331→169]: 125  [331→271]: 253, 211, 193, 169 |  | Galloyl-hexoside |
| **11** | 645 [M-H]^-^  649 [M-H]^-^  633 [M-H]^-^ | 483, 357, 303  631, 497, 301  481, 463, 301, 275, 257 | [645→483]: 439, 357, 303, 237, 177  [649→497]: 301  [633→301]: 284, 257, 229, 185 | [645→483→303]: 275, 207, 177, 151  [649→497→301]: 257, 229, 185, 145 | Unknown  Galloyl-HHDP-gluconic acid  (Lagerstannin C) isomer 1  Galloyl-HHDP-hexoside |
| **12** | 643 [M-H]^-^  645 [M-H]^-^ | 625, 505, 481, 463, 355, 301, 283  483, 357, 303 | [643→463]: 301, 283  [645→483]: 439, 357, 303, 237, 177 | [643→463→283]: 255, 239, 227  [645→483→303]: 275, 207, 177, 151 | Ellagitannin 1  Unknown |
| **13** | 649 [M-H]^-^ | 497, 301 | [649→497]: 301 | [649→497→301]: 257, 229, 185 | Galloyl-HHDP-gluconic acid  (Lagerstannin C) isomer 2 |
| **14** | 368 [M-H]^-^  629 [M-H]^-^ | 324, 188, 179, 161, 144, 131, 119, 113  467, 449, 341, 287 | [368→179]: 161, 149, 143, 131, 119, 113  [629→467]: 423, 357, 341, 287 | [629→467→287]: 259, 243, 219, 215, 203, 177, 175 | Unknown  Unknown |
| **15** | 643 [M-H]^-^ | 625, 481, 463, 355, 301, 283 | [643→481]: 355, 329, 319, 301, 283, 257, 193, 175 | [643→481→301]: 283, 273, 259, 257, 233, 175 | Ellagitannin 2 |
| **16** | 1083 [M-H]^-^ | 1065, 781, 721, 601, 575, 549 | [1083→781]: 721, 665, 601, 575, 299 | [1083→781→601]: 555, 299, 271 | HHDP-gallagyl-hexoside (Punicalagin) isomer 1 |
| **17** | 329 [M-H]^-^ | 311, 269, 239, 209, 167 | [329→167]: 152, 123, 108 |  | Vanillic acid glucoside |
| **18** | 341 [M-H]^-^ | 179 | [341→179]: 135 |  | Caffeic acid hexoside |
| **19** | 479 [M-H]^-^ | 461, 435, 317, 299, 191 | [479→299]: 281, 271, 255, 243 | [479→299→243]: 225, 215, 199 | Unknown |
| **20** | 951 [M-H]^-^ | 907, 783 | [951→907]: 783, 745, 605, 481, 301, 275 | [951→907→783]: 481, 301, 275 | HHDP-valoneoyl-glucoside |
| **21** | 1417 [M-H]^-^  708 [M-2H]^2-^ | 1247, 1115, 1085, 1059, 785, 783, 765, 633, 613, 451, 301  1247, 1085, 783, 765, 633, 613, 301 | [1417→633]: 615, 481, 463, 301, 275, 249  [1417→765]: 747, 721, 613, 597, 427, 301, 275  [1417→785]: 767, 633, 483, 419, 301, 275,249  [708→633]: 615, 481, 301, 275, 249 | [708→633→301]: 257, 229, 185 | Ellagitannin (Camptothin A)* |
| **22** | 481 [M-H]^-^ | 319, 301 |  |  | HHDP- hexoside |
| **23** | 1567 [M-H]^-^  783 [M-2H]^2-^ | 1549, 1397, 1209, 935, 915, 783, 765, 753  1379, 935, 769, 765, 613, 451, 301, 275, 249 | [1567→765]: 747, 613, 597, 533, 427, 401, 301, 275  [1567→935]: 917, 783, 765, 451, 427, 401, 301, 275 | [1567→935→765]: 747, 703, 597, 533, 427, 401, 399, 301, 275 | Oenothein B isomer 1 |
| **24** | 785 [M-H]^-^  1083 [M-H]^-^ | 767, 633, 615, 483, 463, 419, 331, 301, 275, 249  781, 721, 601, 575 | [785→633]: 615, 463, 421, 301, 275, 249  [785→483]: 331, 313, 295, 169  [785→301]: 257, 229, 185  [1083→781]: 721,  601, 575, 299 | [1083→781→601]: 299, 271 | Digalloyl-HHDP-hexoside isomer (Pedunculagin II) isomer 1  HHDP-gallagyl-hexoside (Punicalagin) isomer 2 |
| **25** | 525 [M-H]^-^ | 363, 315, 167 | [525→363]: 315, 195, 167, 151 | [525→363→167]: 151, 123 | Vanillic acid derivative 1 |
| **26** | 525 [M-H]^-^ | 363, 315, 195, 167 | [525→363]: 315, 195, 167, 151 |  | Vanillic acid derivative 2 |
| **27** | 1085 [M-H]^-^  1567 [M-H]^-^  783 [M-2H]^2-^ | 783, 765, 633, 597  1549, 1265, 1247, 1085, 935, 933, 915, 785, 783, 765, 763  1379, 1265, 935, 933, 769, 765, 633, 481, 301 | [1085→765]: 613, 597, 595, 301, 275  [1567→765]: 613, 597, 533, 401, 301  [783→765]: 747, 613, 597, 533, 463, 427, 399, 301, 275 |  | Tri-HHDP-hexoside  Oenothein B isomer 2 |
| **28** | 1085 [M-H]^-^  1567 [M-H]^-^  783 [M-2H]^2-^ | 783, 765, 633, 597  1549, 1265, 1247, 1085, 935, 933, 915, 785, 783, 765, 763  1265, 935, 633, 613, 481, 301 | [1085→765]: 613, 597, 595, 301, 275  [1567→765]: 613, 597, 533, 401, 301 |  | Tri-HHDP-hexoside  Oenothein B isomer 3 |
| **29** | 785 [M-H]^-^  643 [M-H]^-^ | 767, 633, 615, 483, 463, 419, 331, 301, 275, 249  625, 463, 481, 345 | [785→633]: 615, 463, 421, 301, 275, 249  [785→483]: 331, 313, 295, 169  [785→301]: 257, 229, 185  [643→481]: 463,345, 315, 255, 165, 153 | [643→481→345]: 327, 183, 165, 139  [643→481→315]: 255, 195, 177, 153 | Digalloyl-HHDP-hexoside isomer (Pedunculagin II) isomer 2  Ellagitannin 3 |
| **30** | 341 [M-H]^-^ | 179, 161, 135 |  |  | Caffeic acid hexoside isomer |
| **31** | 463 [M-H]^-^  555 [M-H]^-^ | 301  537, 393, 197 | [463→301]: 257, 229, 185, 145  [555→393]: 375, 345, 197, 195, 182, 165, 151 | [555→393→197]: 182, 153 | Ellagic acid hexoside  Syringic acid derivative |
| **32** | 551 [M-H]^-^ | 491, 461, 431, 389, 341, 193 | [551→389]: 341, 193, 165, 149, 134 | [551→389→193]: 178, 149, 134  [551→389→341]: 326, 309, 297, 282, 265, 250, 235, 233, 205 | Ferulic acid-*C*-hexoside  derivative |
| **33** | 785 [M-H]^-^ | 767, 633, 615, 483, 463, 419, 331, 301, 275, 249 | [785→615]: 571, 445, 419, 301, 275  [785→419]: 401, 359, 329, 293, 275, 249, 219  [785→301]: 257, 229, 185 |  | Digalloyl-HHDP-hexoside isomer (Pedunculagin II) isomer 3 |
| **34** | 1569 [M-H]^-^  784 [M-2H]^2-^ | 1550, 1531, 1399, 1247, 1085, 935, 785, 765, 597, 451  1085, 935, 785, 765, 699, 633, 623, 529, 483, 419, 301, 275, 249 | [1569→765]: 747, 721, 613, 597, 533, 427, 401, 301, 275  [1569→935]: 917, 891, 811, 783, 765, 613, 451, 425, 327  [1569→785]: 767, 757, 633, 615, 533, 483, 419, 301, 275, 249  [784→765]: 915, 766, 721, 613, 597, 553, 533, 445, 427, 301, 275  [784→935]: 936, 917, 811, 783, 765, 755, 739, 696, 613, 487, 483, 451, 425, 367, 301 | [1569→765→597]: 579, 553, 509, 445, 427, 401, 355, 301, 275, 249  [1569→935→451]: 433, 405, 395, 377, 327, 301  [784→765→597]: 553, 535, 509, 445, 427, 401, 383, 325, 301, 275 | Eucalbanin B isomer 1 |
| **35** | 1569 [M-H]^-^  784 [M-2H]^2-^ | 1550, 1531, 1399, 1247, 1085, 935, 785, 765, 597, 451  1085, 935, 785, 765, 699, 633, 623, 529, 483, 419, 301, 275, 249 | [1569→765]: 747, 721, 613, 597, 533, 427, 401, 301, 275  [1569→935]: 917, 891, 811, 783, 765, 613, 451, 425, 327  [1569→785]: 767, 757, 633, 615, 533, 483, 419, 301, 275, 249  [784→765]: 915, 766, 721, 613, 597, 553, 533, 445, 427, 301, 275  [784→935]: 936, 917, 811, 783, 765, 755, 739, 696, 613, 487, 483, 451, 425, 367, 301 | [1569→765→597]: 579, 553, 509, 445, 427, 401, 355, 301, 275, 249  [1569→935→451]: 433, 405, 395, 377, 327, 301  [784→765→597]: 553, 535, 509, 445, 427, 401, 383, 325, 301, 275 | Eucalbanin B isomer 2 |
| **36** | 1176 [M-2H]^2-^  784 [M-3H]^3-^  951 [M-H]^-^  953 [M-H]^-^  429 [M-H]^-^ | 1869, 1719, 1569, 1549, 1417, 1399, 1247, 1091, 935, 785, 765, 633, 615, 533, 483, 463, 451, 419  1399, 1267, 1085, 935, 765, 633, 615, 529, 483, 301, 275  933, 915, 765, 613, 445, 301  935, 917, 909, 891, 853, 785, 633, 463, 343, 301  325, 307, 265, 235, 163 | [1176→1569]: 1913, 1398, 1379, 1245, 1076, 935, 786, 598, 451  [784→765]: 916, 766, 721, 613, 597, 553, 533, 445, 427, 401, 301, 275  [784→301]: 257, 299, 185  [951→933]: 915,  897, 765, 631, 613, 463, 445, 301, 275  [953→935]: 917, 899, 765, 633, 615, 463, 445, 343, 301, 275  [429→163]: 145, 127, 119, 103, 89 | [784→765→597]: 553, 509, 445, 427, 399, 301, 275  [951→933→301]: 257, 229  [953→935→301]: 257, 229 | Eucarpanin T1 isomer 1  Galloyl-HHDP-DHHDP-hexoside (Granatin B)  Galloyl-chebuloyl-HHDP-glucose  (Chebulagic acid)  Coumaric acid derivative |
| **37** | 1176 [M-2H]^2-^  784 [M-3H]^3-^  447 [M-H]^-^  433[M-H]^-^ | 1869, 1719, 1569, 1549, 1417, 1399, 1247, 1091, 935, 785, 765, 633, 615, 533, 483, 463, 451, 419  1399, 1267, 1085, 935, 765, 699, 633, 615, 543, 483, 301  300, 301  301 | [784→765]: 766, 721, 613, 597, 553, 533, 443, 427, 401,  301, 275  [447→301]: 257, 229  [433→301]: 257, 229, 185 |  | Eucarpanin T1 isomer 2  Ellagic acid-deoxyhexoside  Ellagic acid-pentoside |
| **38** | 301 M-H]^-^ | 257, 229, 185 |  |  | Ellagic acid |
| **39** | 1176 [M-2H]^2-^  784 [M-3H]^3-^ | 1869, 1719, 1569, 1549, 1417, 1399, 1247, 1091, 935, 785, 765, 633, 615, 533, 483, 463, 451  1399, 1267, 1085, 935, 765, 699, 633, 615, 543, 483, 301 | [784→765]: 766, 721, 613, 597, 553, 533, 443, 427, 401, 301, 275 |  | Eucarpanin T1 isomer 3 |
| **40** | 507 [M-H]^-^ | 489, 345, 327, 315, 209 | [507→327]: 312, 296, 283  [507→345]: 327, 315, 301, 221, 209 |  | Pomegralignan |
| **41** | 937 [M-H]^-^ | 919, 893, 785, 767, 635, 617, 597, 483, 465, 445, 419, 313, 301, 275, 257 |  |  | Trigalloyl-HHDP-hexoside |
| **42** | 533 [M-H]^-^ | 515, 503,473, 425, 395, 371, 353, 341 | [533→353]: 338 |  | Guaiacyl(8-5)ferulic acid hexoside |

* Tentatively identified based on the mass spectral data cited by [58]

Abbreviations used: HHDP, hexahydroxydiphenoyl; DHHDP, dehydrohexahydroxydiphenoyl
