# Supplementary material for: Qualitative and biochemical characteristics of pomegranate fruit grown using reclaimed water and low input fertigation treatments at harvest and during storage
Source: Heliyon. 2024 Jul 14;10(14):e34430. doi: 10.1016/j.heliyon.2024.e34430 (PMC11315205; doi:10.1016/j.heliyon.2024.e34430)
Supplement: Multimedia component 3 [file mmc3.docx]

**Table S3**

Concentration of individual polyphenols of pomegranate samples freshly picked and grown using conventional water + conventional fertigation (CW-CF) determined by HPLC-UV/Vis. Data are mean of 3 replicates ± standard deviation.

| ***Phenolic Compounds*** | | ***mg 100g****^-1^* ***fw*** | | |
| --- | --- | --- | --- | --- |
| 1 | Delphinidin 3,5-diglucoside | 7.83 | ± | 0.89 |
| 2 | Cyanidin 3,5-diglucoside | 4.70 | ± | 0.53 |
| 3 | Delphinidin 3-glucoside | 7.29 | ± | 1.91 |
| 4 | Cyanidin 3-glucoside | 4.40 | ± | 1.09 |
| 5 | Pelargonidin 3-glucoside | 0.21 | ± | 0.08 |
| 6 | Cyanidin 3-pentoside1 | 0.21 | ± | 0.06 |
| 7 | Cyanidin 3-pentoside2 | 0.13 | ± | 0.03 |
| 10 | Galloyl hexoside | 6.85 | ± | 0.24 |
| 11 | Galloyl-HHDP-gluconic acid (Lagerstannin C) isomer 1+ Galloyl-HHDP-hexoside + Unknown | 2.82 | ± | 0.28 |
| 12 | Ellagitannin 1 + Unknown | 1.96 | ± | 0.21 |
| 13 | Galloyl-HHDP-gluconic acid (Lagerstannin C) isomer 2 | 3.79 | ± | 0.50 |
| 15 | Ellagitannin 2 | 2.74 | ± | 0.48 |
| 16 | HHDP-gallagyl-hexoside (Punicalagin) isomer 1 | 2.85 | ± | 1.80 |
| 17 | Vanillic acid glucoside | 2.55 | ± | 0.95 |
| 18 | Caffeic acid hexoside | 5.05 | ± | 2.52 |
| 20 | HHDP-valoneoyl-glucoside | 10.07 | ± | 4.06 |
| 21 | Ellagitannin (Camptothin A)* | 3.82 | ± | 0.44 |
| 22 | HHDP- hexoside | 1.76 | ± | 0.71 |
| 23 | Oenothein B isomer 1 | 20.61 | ± | 2.45 |
| 24 | Digalloyl-HHDP-hexoside isomer (Pedunculagin II) isomer 1 + HHDP-gallagyl-hexoside (Punicalagin) isomer 2 | 21.46 | ± | 0.93 |
| 25 | Vanillic acid derivative 1 | 10.53 | ± | 1.10 |
| 26 | Vanillic acid derivative 2 | 4.88 | ± | 1.15 |
| 27 | Tri-HHDP-hexoside + Oenothein B isomer 2 | 4.37 | ± | 0.28 |
| 28 | Tri-HHDP-hexoside + Oenothein B isomer 3 | 2.28 | ± | 0.23 |
| 29 | Digalloyl-HHDP-hexoside isomer (Pedunculagin II) isomer 2 + Ellagitannin 3 | 14.84 | ± | 1.95 |
| 30 | Caffeic acid hexoside isomer | 1.87 | ± | 0.11 |
| 31 | Ellagic acid hexoside + Syringic acid derivative | 6.00 | ± | 0.76 |
| 32 | Ferulic acid-*C*-hexoside derivative | 2.07 | ± | 0.37 |
| 33 | Digalloyl-HHDP-hexoside isomer (Pedunculagin II) isomer 3 | 2.69 | ± | 0.33 |
| 34 | Eucalbanin B isomer 1 | 6.70 | ± | 0.50 |
| 35 | Eucalbanin B isomer 2 | 16.30 | ± | 2.17 |
| 36 | Eucarpanin T1 isomer 1 + Galloyl-HHDP-DHHDP-hexoside (Granatin B) + Galloyl-chebuloyl-HHDP-glucose (Chebulagic acid) + Coumaric acid derivative | 14.71 | ± | 1.16 |
| 37 | Eucarpanin T1 isomer 2 + Ellagic acid-deoxyhexoside + Ellagic acid-pentoside | 10.00 | ± | 0.59 |
| 38 | Ellagic acid | 1.71 | ± | 0.63 |
| 39 | Eucarpanin T1 isomer 3 | 3.98 | ± | 0.16 |
| 41 | Trigalloyl-HHDP-hexoside | 2.60 | ± | 0.40 |
| 42 | Guaiacyl(8-5)ferulic acid hexoside | 0.96 | ± | 0.11 |
|  |  |  |  |  |

* Tentatively identified based on the mass spectral data cited by [58]

Abbreviations used: HHDP, hexahydroxydiphenoyl; DHHDP, dehydrohexahydroxydiphenoyl
